# Sociodemographic characteristics of patients and their use of post-bariatric contouring surgery in the US

**DOI:** 10.1186/s12913-022-07692-1

**Published:** 2022-03-07

**Authors:** Ibrahim Al-Sumaih, Michael Donnelly, Ciaran O’Neill

**Affiliations:** 1 Centre for Public Health, School of Medicine, Dentistry and Biomedical Sciences, Institute of Clinical Sciences, Queen’s University Belfast, Block B, Royal Victoria Hospital, Belfast, UK BT12 6BA UK; 2 Ministry of Health, Riyadh, Saudi Arabia

**Keywords:** Insurance coverage, Ambulatory surgical procedures, United States, Delivery of health care

## Abstract

**Background:**

Expansion of healthcare insurance coverage to bariatric surgeries has led to an increased demand from patients for post-bariatric contouring surgeries. This study examined the relationship between the use of contouring procedures on post-bariatric surgery patients, clinical need and sociodemographic factors.

**Methods:**

Data were extracted from the Healthcare Cost and Utilization Project (HCUP) Nationwide Ambulatory Surgery Sample (NASS) regarding hospital-owned ambulatory surgical centres in the US. Episodes without missing values relating to patients, 18 years old or above were the primary unit of analysis. Episodes were excluded if the field regarding expected payer was reported as “no charge.” The primary outcome was the likelihood of panniculectomy, abdominoplasty, and mastopexy among post-bariatric surgery patients; and the degree to which uptake of these types of contouring surgery were associated with age, gender, median households’ income, expected payer, patient’s location, and comorbidity.

**Results:**

A total of 66,368 weighted episodes of care received by patients who had had bariatric surgery were extracted (54,684 female [82.4%]; mean age, 51.3 [SD, 12.1]; 6219 episodes had contouring surgeries [9.37%]). Panniculectomy was the most common post-bariatric contouring procedure (3.68%). Uptake of post-bariatric contouring procedures was associated with age, sex, payment type, area-based measures of median household income, and patient location. Compared to Medicare insured patients, the odds of receiving contouring surgery among self-payers were 1.82 (95% CI, 1.47 to 2.26) for panniculectomy, 14.79 (95% CI, 12.19 to 17.93) for abdominoplasty and 47.97 (95% CI, 32.76 to 70.24) for mastopexy. Rank order of comorbidity profiles also differed between insured and self-paying recipients of contouring surgery.

**Conclusions:**

Insurance status of bariatric surgery patients and their sex were strongly associated with receipt of a range of contouring procedures. Self-payments were associated with a doubling of the odds of having panniculectomy and an increase in the odds to approximately 14 times for abdominoplasty and 48 times for mastopexy. Thus, access to contouring surgery by post-bariatric patients may be disproportionately dependent on personal preference supported by ability to pay rather than clinical need. Further research is needed to examine the impact of contouring or delayed/denied contouring on health status.

**Supplementary Information:**

The online version contains supplementary material available at 10.1186/s12913-022-07692-1.

## Background

Obesity is a global epidemic [1]. In the US, the prevalence of obesity had reached 38.5% in 2016 and is expected to affect 44% of the US population by 2031 [2]. The direct medical cost of obesity and obesity-related diseases was estimated at $98.1 billion in the US in 2008 [3]. Numerous weight reduction interventions have been attempted to address the epidemic but bariatric surgery remains the most cost-effective and durable intervention at an individual level [4, 5]. Bariatric surgery has become more prevalent in the US after insurance coverage expansion to include laparoscopic sleeve gastrectomy in 2011 [6]. Patients are expected to reach their lowest weight in the first year after bariatric surgery [7]. Health concerns as well as self-image have been posited as the main motivators among bariatric surgery candidates [8–10]. The rapid weight loss post-surgery, however, can lead to sagging skin in different parts of the body [11]. Patients commonly complain of sagging skin at the anterior abdomen and chest which can result in poor hygiene, recurrent infection, impaired: mobility; sexual relations; social interaction; quality of life and; negative self-image [11–14].

Contouring surgery may improve the post-bariatric patient’s self-image, quality of life and help address conditions associated with the sagging skin [15]. It is also argued to play a role in maintaining body weight and prevent weight regain after bariatric surgery [16, 17]. The rate of contouring surgeries among those who lost significant weight in the US grew by 10% from 2015 to 2016 [18]. In the US, more than two thirds of the post-bariatric population desire to have contouring surgeries but cost remains a key barrier [19]. The American Society of Plastic Surgeons have recommended criteria for classification of contouring procedures into reconstructive and cosmetic procedures in order to encourage insurance expansion and enhance patients access to reconstructive procedures like panniculectomy and mastopexy [20, 21]. The availability of ambulatory surgical centres has also facilitated increased patient access to most elective surgeries by lowering cost including abdominoplasty which is principally (48.8%) performed in ambulatory surgical centers [22, 23].

There is a paucity of research examining the post-bariatric patient’s access to, and use of, contouring procedures. Among the few studies in this area, one in New York State, US (a state which was considered to be flexible in terms of criteria for reimbursement) found that only 6% of post-bariatric patients had undergone contouring surgeries [24, 25]. Another recent study on a smaller sample found that 12.7% of bariatric population underwent contouring surgery in the US [26]. Giladi et al. [27] investigated Medicaid expansion which included panniculectomy on New York patient’s access to contouring surgery and found that an increasing number of Medicaid patients accessed panniculectomy but the upward utilisation trend by uninsured patients was not affected. The rising trend of uninsured bariatric patients presenting for contouring suggests a divergence between patients’ wants and insurance company criteria based on clinical need. Studies have highlighted a disconnect between the opinions of Members of the American Society of Plastic Surgeons on the grounds for which access to contouring should be provided and the criteria used by insurance companies [25]. For example, conditions such as depression and anxiety, commonly found in the post-bariatric population may not provide a basis upon which insurance coverage is offered [28, 29].

Given the current paucity of research, this study (the first study of its type using nationally representative data from hospital-owned ambulatory surgical centres) investigated factors that may contribute to differences in uptake of contouring surgeries among post-bariatric surgery patients in the US.

## Methods

### Data source

The study data were drawn from 2016 discharge data from the Nationwide Ambulatory Surgery Sample (NASS), Healthcare Cost and Utilization Project (HCUP), Agency for Healthcare Research and Quality. The HCUP NASS data is the largest ambulatory care dataset in the US and includes 7,608,879 observations from 63% of hospital-owned ambulatory surgical facilities in the US [30]. The data included the age and gender as well as median household income for the ZIP code of the patient whose episode was captured in the data. Also included were details of the expected payer, total charge, the Current Procedural Terminology (CPT) codes for procedures performed, tenth revision of the International Classification of Diseases (ICD 10) codes for patient’s diagnoses, and facility characteristics including bed size capacity, hospital location and teaching status at which services were provided.

### Population

Data for episodes of care where bariatric surgery (ICD 10 code is Z9884) appeared on the list of co-morbidities were included in the study. Episodes that related to a person less than 18 years old or having a missing value among included variables were excluded. To avoid potential disclosure issues episodes where the expected payer was reported as “no charge” were also excluded (15 episodes). CPT codes for post-bariatric contouring procedures were adopted from the American Society of Plastic Surgeons guidelines for recording contouring procedures [31]. A full list of CPT codes of post-bariatric contouring procedures used in the study can be seen in (Additional file 1: Table A1). Forearm and submental skin excision were merged with excision of excessive skin in other areas in order to adhere to the HCUP guidelines for reporting observations of equal or less than 10 observations per cell [32]. For the purpose of exploring common medical history that bariatric population present with, a list of the common ICD 10 was generated for each of the popular contouring procedures. A full list of the ICD 10 codes used can be seen in (Additional file 1: Table A2).

### Statistical analysis

Statistical analysis of the weighted and unweighted sample was presented as frequency and percentages for categorical variables as well as mean and standard deviation for continuous variables. Total charges were reported as medians and interquartile ranges because of the skewness (See Additional file 1: Table A3). The results of the weighted sample analyses are those presented. All results were weighted according to the recommendations by the HCUP [33]. Frequencies of the weighted variables were rounded. Logistic regression was used to estimate the odds ratios and 95% confidence intervals for covariates in regressions related to panniculectomy, abdominoplasty and mastopexy. In order to examine variance in possession of insurance and common medical necessity, analyses also compared uptake based on insurance status using chi square tests. Statistical significance was defined at *p* value < .05. All analyses were undertaken in Stata version 15 (StataCorp LLC College Station, TX).

## Results

A total of 66,368 episodes of post-bariatric patients were extracted. The majority of the sample was female (82.40%), and the mean age was approximately 51 years old. More than half of the bariatric sample held private insurance (37,561; 56.60%) and a minority were self-payers (2359; 2.89%). The contouring procedure was performed on approximately one tenth of the post-bariatric sample (9.37%). Panniculectomy (3.68%) and abdominoplasty (3.51%) were the most common contouring procedures among the bariatric population followed by mastopexy (1.16%). Detailed descriptive statistics of the sample are presented in Tables 1 and 2. Median total charges of mastopexy, panniculectomy, and abdominoplasty were 27,130, 25,178, and 24,656 US dollar, respectively (See Additional file 1: Table A1).

**Table 1:** Descriptive statistics of the sample

Variable	Unweighted (***n*** = 48,263)	Weighted (***n*** = 66,368)
Female, No. (%)	39,739 (82.34)	54,684 (82.40)
Age, mean (SD), years	51.29 (12.11)	51.30 (12.12)
Expected payer, No. (%)		
Medicare	11,960 (24.78)	16,345 (24.63)
Medicaid	5544 (11.49)	7520 (11.33)
Private	27,182 (56.32)	37,561 (56.60)
Self-pay	1685 (3.49)	2359 (3.55)
Other	1892 (3.92)	2582 (3.89)
Income, No. (%)		
Highest income quartile	10,556 (21.96)	14,642 (22.06)
Second highest income quartile	13,468 (27.91)	18,422 (27.76)
Second lowest income quartile	13,160 (27.27)	18,028 (27.16)
Lowest income quartile	11,079 (22.96)	15,275 (23.02)
Patient location, No. (%)		
Central counties of more than 1 million population	12,870 (26.67)	17,525 (26.41)
Fringe counties of more than 1 million population	12,266 (25.41)	16,850 (25.39)
Metropolitan of less than 1 million population	11,033 (22.86)	15,193 (22.89)
Metropolitan of less than 250,000 population	4451 (9.22)	5960 (8.98)
Micropolitan	4721 (9.78)	6705 (10.10)
Other	2922 (6.05)	4134 (6.23)
Urban hospital, No. (%)	44,598 (92.41)	60,719 (91.49)
Teaching hospital, No. (%)	34,293 (71.05)	45,639 (68.77)
Hospital bed size capacity, No. (%)		
300+	26,974 (55.89)	35,608 (53.65)
100 – 299	17,089 (35.41)	23,763 (35.81)
< 100	4200 (8.70)	6996 (10.54)
Contouring procedure, No. (%)	4604 (9.54)	6219 (9.37)
Panniculectomy	1802 (3.73)	2443 (3.68)
Abdominoplasty	1739 (3.60)	2327 (3.51)
Modified abdominoplasty	165 (0.34)	229 (0.34)
Excision of thigh skin	296 (0.61)	400 (0.60)
Excision of leg skin	26 (0.05)	32 (0.05)
Excision of hip skin	47 (0.10)	63 (0.10)
Excision of buttock skin	78 (0.16)	105 (0.16)
Excision of arm skin	484 (1.00)	653 (0.98)
Excision of skin in other area	215 (0.45)	289 (0.44)
Mastopexy	559 (1.16)	773 (1.16)
Mastectomy for gynecomastia	31 (0.06)	42 (0.06)

Frequency of weighted variables are rounded

**Table 2:** Detailed descriptive statistics by type of surgery

Variable	Panniculectomy	Abdominoplasty	Mastopexy	Other types of contouring surgeries	None
Female, No. (%)	2116 (86.63)	2074 (89.11)	Males are less than 10	1364 (90.29)	49,149 (81.71)
Age, mean (SD), years	47.84 (11.63)	45.78 (10.96)	45.76 (10.87)	47.92 (11.32)	51.73 (12.12)
Expected payer, No. (%)					
Medicare	598 (24.48)	266 (11.45)	48 (6.22)	137 (9.05)	15,354 (25.53)
Medicaid	448 (18.33)	390 (16.77)	57 (7.43)	189 (12.53)	6508 (10.82)
Private	1107 (45.32)	955 (41.04)	206 (26.66)	451 (29.84)	35,075 (58.31)
Self-pay	200 (8.18)	631 (27.13)	428 (55.39)	653 (43.25)	875 (1.45)
Other	90 (3.68)	84 (3.61)	33 (4.3)	81 (5.33)	2336 (3.88)
Income, No. (%)					
Highest income quartile	451 (18.47)	558 (23.98)	247 (31.98)	423 (28.03)	13,221 (21.98)
Second highest income quartile	668 (27.33)	622 (26.73)	217 (28.13)	427 (28.30)	16,706 (27.77)
Second lowest income quartile	683 (27.94)	547 (23.52)	172 (22.27)	355 (23.47)	16,477 (27.39)
Lowest income quartile	642 (26.26)	600 (25.77)	136 (17.63)	305 (20.20)	13,744 (22.85)
Patient location, No. (%)					
Central counties of more than 1 million population	748 (30.62)	748 (32.13)	249 (32.28)	489 (32.38)	15,534 (25.83)
Fringe counties of more than 1 million population	570 (23.34)	666 (28.62)	236 (30.55)	479 (31.73)	15,163 (25.21)
Metropolitan of less than 1 million population	582 (23.81)	479 (20.59)	149 (19.25)	295 (19.55)	13,849 (23.03)
Metropolitan of less than 250,000 population	188 (7.69)	176 (7.54)	63 (8.20)	97 (6.44)	5508 (9.16)
Micropolitan	204 (8.36)	156 (6.72)	43 (5.61)	72 (4.75)	6275 (10.43)
Other	151 (6.18)	102 (4.39)	31 (4.12)	78 (5.14)	3820 (6.35)
Urban hospital, No. (%)	2335 (95.58)	2249 (96.62)	758 (98.06)	1473 (97.50)	54,713 (90.96)
Teaching hospital, No. (%)	1951 (79.85)	1772 (76.15)	597 (77.33)	1198 (79.32)	40,772 (67.79)
Hospital bed size capacity, No. (%)					
300+	1612 (66.00)	1406 (60.42)	464 (60.08)	944 (62.49)	31,701 (52.70)
100 – 299	648 (26.52)	744 (31.97)	246 (31.79)	478 (31.63)	21,900 (36.41)
< 100	183 (7.48)	177 (7.61)	63 (8.14)	89 (5.88)	6548 (10.89)

Frequency of weighted variables are rounded

The results of logistic regression analysis are shown in Table 3. As can be seen, episodes related to younger and female post-bariatric patients were more likely to have contouring surgeries. Compared to the lowest median household income ZIP code, the highest income zip code dwellers was associated with decreased odds of having panniculectomy (odds ratio [OR], 0.76 [95% CI, 0.65 to 0.89] p .001) and abdominoplasty (OR, 0.78 [95% CI, 0.67 to 0.91] p .002) and increased odds of having mastopexy (OR, 1.44 [95% CI, 1.08 to 1.91] p .013). Compared to Medicare, episodes involving self-payers were associated with higher odds of having panniculectomy (OR, 1.82 [95% CI, 1.47 to 2.26] *p* < 0.001), abdominoplasty (OR, 14.78 [95% CI, 12.19 to 17.93] *p* < 0.001), and mastopexy (OR, 47.97 [95% CI, 32.76 to 70.23] *p* < 0.001). Patients’ geographic locations were also found to be associated with uptake of contouring surgeries with lower odds for episodes involving patients from less densely populated areas compared to more densely populated areas.

**Table 3:** Logistic regression models of common contouring surgeries as outcomes of socioeconomic characteristics

Variable	Panniculectomy	Abdominoplasty	Mastopexy
Age			
18–35	Ref	Ref	Ref
36–45, OR (95% CI)	0.83 (0.71 to 0.96)	0.80 (0.69 to 0.93)	0.85 (0.66 to 1.11)
*P* value	.016	.005	.246
46–55, OR (95% CI)	0.75 (0.64 to 0.87)	0.67 (0.57 to 0.78)	0.64 (0.49 to 0.85)
*P* value	<.001	<.001	.002
56+, OR (95% CI)	0.47 (0.39 to 0.56)	0.38 (0.32 to 0.45)	0.48 (0.36 to 0.65)
*P* value	<.001	<.001	<.001
Female, OR (95% CI)	1.24 (1.08 to 1.43)	1.36 (1.16 to 1.59)	14.96 (6.67 to 33.59)
*P* value	.003	<.001	<.001
Median household income			
Lowest income	Ref	Ref	Ref
Second lowest income, OR (95% CI)	0.96 (0.84 to 1.09)	0.77 (0.67 to 0.89)	1.05 (0.79 to 1.39)
*P* value	.515	<.001	.750
Second highest income, OR (95% CI)	0.91 (0.79 to 1.04)	0.80 (0.69 to 0.93)	1.22 (0.93 to 1.61)
*P* value	.178	.002	.158
Highest income, OR (95% CI)	0.76 (0.65 to 0.89)	0.78 (0.67 to 0.91)	1.44 (1.08 to 1.91)
*P* value	.001	.002	.013
Expected payer			
Medicare	Ref	Ref	Ref
Medicaid, OR (95% CI)	1.10 (0.93 to 1.30)	1.94 (1.58 to 2.37)	1.62 (1.01 to 2.59)
*P* value	.277	<.001	.045
Private, OR (95% CI)	0.62 (0.54 to 0.71)	1.11 (0.93 to 1.32)	1.32 (0.89 to 1.96)
*P* value	<.001	.245	.171
Self-pay, OR (95% CI)	1.82 (1.47 to 2.26)	14.78 (12.19 to 17.93)	47.97 (32.76 to 70.23)
*P* value	<.001	<.001	<.001
Other, OR (95% CI)	0.77 (0.58 to 1.01)	1.51 (1.13 to 2.03)	3.36 (1.99 to 5.66)
*P* value	.055	.006	<.001
Patient location			
Central, OR (95% CI)	Ref	Ref	Ref
Fringe> 1 mln, OR (95% CI)	0.83 (0.73 to 0.96)	0.92 (0.81 to 1.05)	0.79 (0.63 to 1.001)
*P* value	.009	.219	.051
250 K - 1 mln, OR (95% CI)	0.88 (0.77 to 1.00)	0.78 (0.67 to 0.90)	0.80 (0.62 to 1.04)
*P* value	.049	.001	.098
50 K - 250 K, OR (95% CI)	0.71 (0.58 to 0.85)	0.71 (0.58 to 0.87)	0.82 (0.59 to 1.16)
*P* value	<.001	.001	.267
Micropolitan, OR (95% CI)	0.67 (0.55 to 0.81)	0.58 (0.47 to 0.72)	0.62 (0.42 to 0.93)
*P* value	<.001	<.001	.019
Other, OR (95% CI)	0.78 (0.63 to 0.97)	0.56 (0.43 to 0.72)	0.63 (0.40 to 1.00)
*P* value	0.026	<.001	.048

In Tables 4, 5, and 6 common diagnoses among those in receipt of panniculectomy, abdominoplasty, and mastopexy are shown together with insurance status. As can be seen, different patterns are evident in term of the rank ordering of conditions and the insurance status of those receipt of contouring surgery. While self-pay comprised roughly 10% of panniculectomy episodes, it made up over 50% in mastopexy episodes. Similarly, while there is evident similarity in the comorbidities recorded, the rank ordering of these differs markedly.

**Table 4:** Common diagnosis appeared with panniculectomy episodes

Diagnosis	Overall (***n*** = 2443)	Insured (***n*** = 2243)	Self-payer (***n*** = 200)	***P*** value
Pannus, No. (%)	1528 (62.53)	1461 (65.11)	67 (33.60)	<.001
Hypertension, No. (%)	884 (36.20)	828 (36.20)	57 (28.27)	.0427
Panniculitis, No. (%)	572 (23.41)		Small number	<.001
GERD, No. (%)	534 (21.84)	511 (22.78)	23 (11.27)	.0020
Erythema Intertrigo, No. (%)	451 (18.46)		Small number	<.001
Nicotine dependence, No. (%)	443 (18.14)	412 (18.35)	32 (15.76)	.4543
Type 2 Diabetes, No. (%)	422 (17.25)	396 (17.67)	25 (12.60)	.1217
Long term medication, No. (%)	395 (16.15)	369 (16.45)	26 (12.81))	.2852
Obesity, No. (%)	344 (14.08)	323 (14.42)	21 (10.33)	.1987
Depression, No. (%)	316 (12.94)	303 (13.52)	13 (6.45)	.0210

**Table 5:** Common diagnosis appeared with abdominoplasty episodes

Diagnosis	Overall (***n*** = 2327)	Insured (***n*** = 1696)	Self-payer (***n*** = 631)	***P*** value
Pannus, No. (%)	1392 (59.81)	1098 (64.73)	294 (46.60)	<.001
Hypertension, No. (%)	650 (27.92)	525 (30.93)	125 (19.84)	<.001
Lipodystrophy, No. (%)	482 (20.69)	293 (17.27)	189 (29.88)	<.001
GERD, No. (%)	448 (19.25)	347 (20.46)	101 (16.01)	.0428
Cosmetic, No. (%)	405 (17.42)	157 (9.26)	248 (39.34)	<.001
Erythema Intertrigo, No. (%)	358 (15.38)	328 (19.36)	30 (4.69)	<.001
Nicotine dependence, No. (%)	356 (15.32)	278 (16.40)	78 (12.40)	.0434
Panniculitis, No. (%)	326 (14.02)	300 (17.72)	26 (4.09)	<.001
Long term medication, No. (%)	283 (12.16)	199 (11.74)	84 (13.28)	.3973
Obesity, No. (%)	275 (11.80)	235 (13.84)	40 (6.31)	<.001

**Table 6:** Common diagnosis appeared with mastopexy episodes

Diagnosis	Overall (***n*** = 773)	Insured (345)	Self-payer (428)	***P*** value
Breast ptosis, No. (%)	558 (72.22)	227 (65.80)	331 (77.39)	.0030
Cosmetic, No. (%)	251 (32.50)	62 (18.02)	189 (44.17)	<.0001
Hypertension, No. (%)	177 (22.97)	107 (30.95)	71 (16.55)	.0001
Lipodystrophy, No. (%)	151 (19.51)	55 (15.95)	96 (22.38)	.0594
Long term medication, No. (%)	132 (17.09)	64 (18.71)	68 (15.79)	.3789
Pannus, No. (%)	132 (17.04)	69 (20.16)	62 (14.52)	.0784
GERD, No. (%)	118 (15.32)	61 (17.71)	57 (13.39)	.1694
Nicotine dependence, No. (%)	107 (13.90)	56 (16.36)	51 (11.93)	.1430
Abnormal weight loss, No. (%)	101 (13.10)	25 (7.36)	76 (17.72)	.0005
Breast hypoplasia, No. (%)	95 (12.35)	29 (8.55)	66 (15.41)	.0148

## Discussion

Sagging skin is a common unwanted outcome of bariatric surgeries that can also give rise to health issues. A majority of the post-bariatric population want surgical contouring but many lack access to their preferred type of surgeries due to high out of pocket costs [34]. The current study estimated that of all inpatient episodes among patients who had previously had bariatric surgery just over 9% were for at least one contouring surgery in 2016. Some caution is warranted in the interpretation of this statistic. As noted, we examine here episodes of care not patients – a small number of patients may have received more than one procedure separately in the same year. Also, we examine episodes of care in 1 year only, i.e. the incidence of contouring not the prevalence, if a patient had received contouring prior to 2016 related to previous bariatric surgery this would not be captured in our data. This said, as noted most contouring takes place within a year of surgery and our estimate of incidence falls within the range of previous estimates of prevalence over a long term period in the US [24, 26] and of prevalence in a recent Dutch study again over a longer time period (9.13%) [17]. We can think of no reason why incident contouring in 2016 would exhibit a different distribution with respect to any of the variables used to explain uptake in 2016 compared to prevalence. Our findings in respect of the relationship with uptake, therefore, remain robust.

The common contouring procedures found in our study were the excision of excess skin in the abdominal area either by panniculectomy or abdominoplasty which account for 7.19% of the total post-bariatric episodes. A previous longitudinal study by Altieri et al. (2017) had examined 37,806 patients underwent bariatric surgeries between 2004 and 2010 in the New York state and found that only 5.58% of the bariatric population had underwent abdominal contouring surgeries within 4 years of having the bariatric surgery [24]. It seems possible that the higher rate of abdominal contouring surgeries found in our study is due to the steady increasing trend of body contouring between 2013 and 2016 by 31% as estimated by the American Society of Plastic Surgeons [18, 35]. Another explanation could be related to the differences in the study design, as Altieri study was confined the length of time since bariatric surgeries to 4 years where in our study participants could had either bariatric or bariatric surgeries at any time in the past.

Among all the socioeconomic variables examined in the logistic regression models, the expectation of self-pay is notable as is being a female. The odds for having contouring procedures among self-payers’ episodes compared to Medicare are 1.82 and 14.79 for panniculectomy and abdominoplasty, respectively. Differences in the odds for different procedures may reflect healthcare insurers’ positions towards contouring surgeries. Recent literature has examined healthcare insurers policies including Medicare and Medicaid toward contouring surgeries coverage. While 98% of healthcare insurers grant cover under certain circumstances for panniculectomy, only 29% of the insurers cover abdominoplasty [36]. The low rate of abdominoplasty coverage may explain the higher odds of self-pay as the only alternative means of accessing the procedure. This is consistent with a perception of the procedure being viewed as elective in nature and difficult to insure given the potential for moral hazard.

The uptake of contouring surgeries is more evident among females as expected. Data from the American Society for Aesthetic Plastic Surgery shows that more than 90% of the cosmetic surgical procedures in 2016 were performed on women [37]. High consumption of abdominal contouring procedure was also established in Altieri [24]. Females were also known to have a higher rate of consumption of bariatric surgeries. In a cross-sectional study on Medicare data, out of 77,774 bariatric surgeries between 2014 and 2016, approximately 74% were performed on females [38].

Insurance coverage for mastopexy is even less common. It has been reported that just 23% of insurers provide coverage for post-bariatric mastopexy including Medicaid [39]. This low level of coverage likely explains the higher dependence on self-pay as a source of funding for this procedure and as with others is likely grounded in its perceived elective nature and the perceived potential for moral hazard by insurers.

Another interesting finding is the pattern of comorbidities between insured and self-payers episodes. The majority of the healthcare insurers provide coverage against certain criteria particularly for panniculectomy and abdominoplasty [36]. The eligibility criteria vary across insurers [25]. In the case of panniculectomy, the commonly presented eligibility criterion is maceration of skin or skin infection not relived by topical or oral medication [25], which would explain the low number (below 10) of erythema intertrigo and panniculitis among self-payers.

Abdominoplasty episodes, on the other hand, are less adherent to the panniculectomy eligibility criteria related to comorbidities. Although it is covered by one-third of insurance companies, most of the insurers would cover abdominoplasty if the patients have diastasis recti. Examining the top common conditions associated with abdominoplasty, however, did not show diastasis recti as only 132 have diastasis recti of which 40% are self-payers. Although the presence of panniculectomy eligibility criteria such as intertrigo and panniculitis have diminished when examining the abdominoplasty episodes, a significant higher proportion of insured candidates have opted to choose abdominoplasty indicated that eligibility for panniculectomy was not limited to intertrigo and panniculitis. Ngaage et al. (2020) have recently examined insurance coverage policies and found that criteria for panniculectomy reimbursement was not limited to comorbidities but also include complaining of impaired mobility, having a minimum duration since bariatric surgery, a significant weight reduction achieved and maintained [36].

Breast ptosis followed by encounter for cosmetic reasons are the most common ICD 10 diagnosis codes for mastopexy episodes. The lack of insurance coverage for mastopexy in post-bariatric population is associated with a significantly higher prevalence of breast ptosis and cosmetic reasons among self-payers compared to insured. Only three private insurance companies have developed eligibility criteria for mastopexy [39]. The criteria include functional impairment, photographed breast ptosis, skin infection, and psychiatric assessment.

Our study has a number of limitations. First, it is based on cross-sectional data which allow us only to look at associations and not draw causal inferences. While this is a limitation imposed on us by the data, it still allows us to describe the activity and the nature of relations in an understudied area. Second, we are limited by the variables available to us to shed light on what may be interesting relationships. We cannot, for example, describe the interval between receipt of bariatric surgery and subsequent contouring surgery.

Similarly, as contouring is an elective procedure, customer choice may have been affected by many variables including surgeon reputation and in-network and out-of-network status as well as common criteria for reimbursement such as weight maintenance duration were not measured in the data.

Another missing variable that might have an impact on the overall rate of contouring surgeries is the history of previously performed contouring surgeries.

Again, these are limitations imposed on us by the data but our results help identify as avenues for further research.

Third, HCUP relates to observed episodes of care, rather than patients per se. It is, therefore, possible that two or more episodes belong to the same patient which could result in some overestimation of the number of procedures and of the degree of difference between some groups. It seems unlikely that instances of multiple procedures in a given year though would materially affect our findings.

Fourth, our data are drawn solely from the US and the findings may not be readily extrapolated to other countries. It is therefore important that these issues including experiences in other countries are addressed in further research.

## Conclusion

Uptake of contouring appears to be significantly related to non-clinical factors such as insurance status and sex. Self-payment is the predominant payment method among those who underwent post-bariatric contouring surgeries indicating potential issues with the operation of insurance in this area that warrant further investigation.

## Supplementary Information

**Additional file 1: Table A1.** CPT codes for contouring surgeries. **Table A2.** ICD 10 codes. **Table A3.** Total charge for solo procedure in US dollar.

## Data Availability

The dataset supporting the conclusions of this article have been obtained from the Healthcare Cost and Utilization Project (HCUP). This dataset is publicly available with restrictions and can be ordered using the following link: https://www.distributor.hcup-us.ahrq.gov/.

## Abbreviations

HCUP: Healthcare Cost and Utilization Project
NASS: Nationwide Ambulatory Surgery Sample
CPT: Current Procedural Terminology
ICD 10: Tenth revision of the International Classification of Diseases
