# Supplementary material for: Sociodemographic characteristics of patients and their use of post-bariatric contouring surgery in the US
Source: BMC Health Serv Res. 2022 Mar 7;22:308. doi: 10.1186/s12913-022-07692-1 (PMC8900300; doi:10.1186/s12913-022-07692-1)
Supplement: Supplementary file 1 — Additional file 1: Table A1. CPT codes for contouring surgeries. Table A2. ICD 10 codes. Table A3. Total charge for solo procedure in US dollar. [file 12913_2022_7692_MOESM1_ESM.docx]

| **Table A1. CPT codes for contouring surgeries** | |
| --- | --- |
| **Procedure** | **Code** |
| Panniculectomy | 15830 |
| Abdominoplasty | 15830 + 15847 |
| Modified abdominoplasty | 17999 |
| Thighplasty | 15832 |
| Excision of excessive leg skin | 15833 |
| Excision of excessive hip skin | 15834 |
| Excision of excessive buttock skin | 15835 |
| Brachioplasty | 15836 |
| Excision of excessive forearm skin | 15837 |
| Excision of excessive submental skin | 15838 |
| Excision of excessive skin of other area | 15839 |
| Mastectomy for gynecomastia | 19300 |
| Mastopexy | 19316 |

| **Table A2. ICD 10 codes** | |
| --- | --- |
| **Condition** | **ICD 10** |
| Pannus | E65 |
| Hypertension | I10 |
| GERD | K219 |
| Panniculitis | M793 |
| Erythema intertrigo | L304 |
| Nicotine dependence | Z87891 |
| Lipodystrophy | E881 |
| Type 2 diabetes mellitus | E119 |
| On medication for long term | Z79899 |
| Obesity | E669 |
| Depression | F329 |
| Breast ptosis | N6481 |
| Abnormal weight loss | R634 |
| Cosmetic reason | Z411 |
| Breast hypoplasia | N6482 |

| **Table A3. Total charge for solo procedure in US dollar** | | | |
| --- | --- | --- | --- |
| **Procedure** | **Weighted sample size** | **Median** | **Interquartile range** |
| Panniculectomy | 1,440 | 25,178 | 17,393 – 36,834 |
| Abdominoplasty | 1,048 | 24,656 | 17,343 – 35,386 |
| Mastopexy | 116 | 27,130 | 18,755 – 41,054 |
